# Cartilaginous endplates: A comprehensive review on a neglected structure in intervertebral disc research

**DOI:** 10.1002/jsp2.1294

**Published:** 2023-10-21

**Authors:** Katherine B. Crump, Ahmad Alminnawi, Paola Bermudez‐Lekerika, Roger Compte, Francesco Gualdi, Terence McSweeney, Estefano Muñoz‐Moya, Andrea Nüesch, Liesbet Geris, Stefan Dudli, Jaro Karppinen, Jérôme Noailly, Christine Le Maitre, Benjamin Gantenbein

**Affiliations:** ^1^ Tissue Engineering for Orthopaedics & Mechanobiology, Bone & Joint Program, Department for BioMedical Research (DBMR), Medical Faculty University of Bern Bern Switzerland; ^2^ Department of Orthopaedic Surgery and Traumatology, Inselspital Bern University Hospital, Medical Faculty, University of Bern Bern Switzerland; ^3^ Graduate School for Cellular and Biomedical Sciences (GCB) University of Bern Bern Switzerland; ^4^ GIGA In Silico Medicine University of Liège Liège Belgium; ^5^ Skeletal Biology and Engineering Research Center, KU Leuven Leuven Belgium; ^6^ Biomechanics Research Unit, KU Leuven Leuven Belgium; ^7^ Twin Research & Genetic Epidemiology St. Thomas' Hospital, King's College London London UK; ^8^ Institut Hospital del Mar d'Investigacions Mèdiques (IMIM) Barcelona Spain; ^9^ Research Unit of Health Sciences and Technology University of Oulu Oulu Finland; ^10^ BCN MedTech, Department of Information and Communication Technologies Universitat Pompeu Fabra Barcelona Spain; ^11^ Division of Clinical Medicine, School of Medicine and Population Health University of Sheffield Sheffield UK; ^12^ Center of Experimental Rheumatology Department of Rheumatology, University Hospital Zurich, University of Zurich Zurich Switzerland; ^13^ Department of Physical Medicine and Rheumatology Balgrist University Hospital, Balgrist Campus, University of Zurich Zurich Switzerland; ^14^ Finnish Institute of Occupational Health Oulu Finland; ^15^ Rehabilitation Services of South Karelia Social and Health Care District Lappeenranta Finland

**Keywords:** biologic therapies, biomechanics, degeneration, pre‐clinical models

## Abstract

The cartilaginous endplates (CEP) are key components of the intervertebral disc (IVD) necessary for sustaining the nutrition of the disc while distributing mechanical loads and preventing the disc from bulging into the adjacent vertebral body. The size, shape, and composition of the CEP are essential in maintaining its function, and degeneration of the CEP is considered a contributor to early IVD degeneration. In addition, the CEP is implicated in Modic changes, which are often associated with low back pain. This review aims to tackle the current knowledge of the CEP regarding its structure, composition, permeability, and mechanical role in a healthy disc, how they change with degeneration, and how they connect to IVD degeneration and low back pain. Additionally, the authors suggest a standardized naming convention regarding the CEP and bony endplate and suggest avoiding the term vertebral endplate. Currently, there is limited data on the CEP itself as reported data is often a combination of CEP and bony endplate, or the CEP is considered as articular cartilage. However, it is clear the CEP is a unique tissue type that differs from articular cartilage, bony endplate, and other IVD tissues. Thus, future research should investigate the CEP separately to fully understand its role in healthy and degenerated IVDs. Further, most IVD regeneration therapies in development failed to address, or even considered the CEP, despite its key role in nutrition and mechanical stability within the IVD. Thus, the CEP should be considered and potentially targeted for future sustainable treatments.

## Graphical abstract

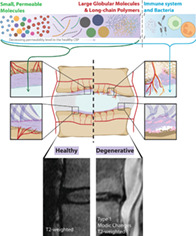

**Abbreviations**
AC: articular cartilage
ACAN: Aggrecan
AF: annulus fibrosus
ALP: alkaline phosphatase
BEP: bony endplate
BMP: bone morphogenetic protein
BV/TV: bone volume fraction
CEP: cartilage endplate
CFD: computational fluid dynamics
COL2: collagen type II gene
ECM: extracellular matrix
eDAPS: endplate‐modified disc‐like angle ply structure
ERK: extracellular signal‐regulated kinase
EZH2: enhancer of zeste homologue 2
FCD: fixed charge density
FE: finite element
FGF: fibroblast growth factor
GAG: glycosaminoglycan
ICD: International Classification of Diseases
IL: interleukin
IVD: intervertebral disc
LBP: low back pain
MAPK: mitogen‐activated protein kinase
MC1/2/3: Modic type 1/2/3 changes
MIF: macrophage migration inhibitory factor
miRNA: microRNA
μ(CT): microcomputed tomography
MMP: matrix metalloproteinase
MRI: magnetic resonance imaging
NP: nucleus pulposus
PEG: polyethylene glycol
PTHrP: parathyroid hormone‐related protein
ROS: reactive oxidative species
Shh: Sonic hedgehog
TEPS: total endplate score
TGF‐β: transforming growth factor beta
TIMP: tissue inhibitors of metalloproteinases
TLR2: toll‐like receptor 2
TLR4: toll‐like receptor 4
UTE: ultrashort time to echo
VB: vertebral body
WT: weight

## 1 INTRODUCTION

### 1.1 What is the CEP?

The intervertebral disc (IVD) provides the spine with flexibility and operational mechanical support. Depending on the speed or dynamism of the mechanical loads, it can store or dissipate energy and allows movement in the vertebral column. The IVD comprises three anatomic regions: a gelatinous core, the nucleus pulposus (NP); the annulus fibrosus (AF), a fibrocartilage that confines the NP laterally; two cartilaginous endplates (CEP) that are thin hyaline‐like cartilage layers, covering the cranial and caudal ends of NP and the inner part of the AF. The human CEP is 0.1–1.6 mm (~0.06 in) thick. It separates the IVD from the adjacent endplates of the vertebral bone, that is, the bony endplates (BEP).^1^, ^2^, ^3^, ^4^ The CEP thickness varies greatly even within healthy IVDs, according to age, location in the spine (disc level), position in the IVD (cranial, caudal), and region in the tissue (central, peripheral). Its extracellular matrix consists of mainly type II collagen, proteoglycans, and water.^5^

The CEP plays a key mechanical role in preventing the disc from bulging into the adjacent vertebral body (VB)^6^ and providing cranial and caudal anchorage for the fibers of the inner AF and NP of the innermost part of the AF (Table 1).^7^, ^8^, ^9^ In addition, the CEP provides a key path for the diffusion of nutrients from the peripheral vasculature to the IVD and waste out of the IVD, which is crucial as it is the largest avascular tissue in the human body.^10^, ^11^ While AC relies on diffusion from the subchondral bone and via synovial fluid for nutrition,^12^, ^13^ the CEP relies on diffusion from neighboring blood vessels. Solutes, including oxygen and glucose, have been hypothesized to be predominantly transported into the disc through the CEP and their availability is regulated by the bone marrow contact channels that cross the BEP.^14^ Often, the combined CEP and BEP are referred to as the vertebral endplate; however, the term vertebral endplate is also used interchangeably to refer purely to the BEP. Although the CEP and BEP have been recognized as distinct tissues since the 1930s, many studies do not distinguish the CEP from the BEP,^15^ for example, when reporting fluid transport in the IVD^16^ or radiological signs of IVD degeneration^17^ even if the authors acknowledge that the vertebral endplate is a bilayer of cartilage and bone.^18^, ^19^ Arguably, it is difficult to isolate the CEP from the BEP experimentally, and this must be done very carefully.^20^, ^21^ Clinically, the distinction of the two tissues on medical images is also very challenging. Thus, when reporting methods, it should be clearly stated what tissue or construct (CEP, BEP, or a combination thereof) is being used and a clear consistent nomenclature should be used, to avoid confusion.^22^ Further, it is the authors' recommendation to define the CEP and the BEP independently where possible, or otherwise explicitly define the vertebral endplate as a construct of two tissues.

**TABLE 1:** Differences between the cartilaginous tissues CEP, AC, NP, and AF regarding: (A) general differences, (B) biochemical composition, and (C) mechanics and permeability.

	Cartilaginous endplate (CEP)	Articular cartilage (AC)	Nucleus pulposus (NP)	Annulus fibrosus (AF)
*A. General*				
Tissue origin	Mesoderm (sclerotome)^26^	Mesoderm^24^, ^25^	Notochord^26^	Mesoderm (sclerotome)^26^
Vascularity	Vascular at birth, avascular in adulthood^26^, ^29^, ^30^	Avascular^13^, ^37^ regardless of the stage of development^39^	Avascular^38^	Vascular at outer one‐third of the AF and avascular at inner AF^36^
Nutrition source	Diffusion from neighboring blood vessels^53^	Diffusion from synovial fluid^12^, ^13^ and the subchondral bone^12^	Anaerobic glycolysis,^51^ diffusion from CEP^50^, ^51^, ^53^	Diffusion from neighboring blood vessels and CEP^50^
Imaging*	Hypointense on T2‐weighted MR indistinguishable from bony endplate. Specific sequences such as UTE assist in differentiating CEP from bony endplate on MR^114^, ^116^, ^222^	Hypointense on T2‐weighted MR and distinguishable from adjacent soft tissue and bone^113^	Hyperintense on T2‐weighted MR, clear delineation from CEP in healthy IVD and gradient in boundary between AF and NP^114^	Hypointense on T2‐weighted MR, indistinguishable from CEP, gradient in boundary between AF and NP in healthy IVD^114^
*B. Biochemical composition*				
Cell density	15 × 10^6^ cells/mL^67^	14–15 × 10^6^ cells/mL^66^	4 × 10^6^ cells/mL^67^	9 × 10^6^ cells/mL^67^
Water content	1.585–1.666 mg water/mg dry wt,^4^ and 22.1%–62.4%^9^, ^60^	70.7%^64^	70%–90%^62^	50%–70%^62^
Proteoglycan	7.2%–13.4% sulfated GAG μg/mg dry weight.^4^ and 4.37%–18.48% μg/mg dry weight^60^	5%–15% GAG by dry weight^61^	30%–50% GAG of dry weight^62^	10% GAG of dry weight^62^
Collagen	681 ± 171 μg/mg dry weight,^4^ and 329.0–886.9 μg/mg dry wt^60^	60%–70% collagen by dry weight^61^	20% of dry weight^62^	70% of dry weight,^62^ aligned with alternate orientations of an average of ±30°^63^
Cell morphology	Rounded and slightly elongated in the direction of the collagen fibers^65^	Superficial and Mid zone: rounded and slightly elongated in the direction of the collagen fibers Deep Zone: round^24^	Fibrochondrocyte‐like cells^76^	Rounded chondrocyte‐like cells (inner AF) and elongated, fusiform, fibroblast‐like cells (outer AF)^77^
Gene markers	ERK, BMP, ACAN, COL1A1, COL2A1^51^	GDF10, CYTL1, IBSP, FBLN1,^78^ ACAN, PTN^79^	PAX1, FOXF1, HBB, CA12, OVOS2,^78^ KRT19,^80^ ACAN, VCAN, TNMD, BASP1, TNFAIP6, FOXF1, FOXF2 and AQP1^79^	COL1, VCAN, PTN, TNMD, BASP1, TNFAIP6, FOXF1, FOXF2, and AQP1^79^
Pericellular matrix	Randomly arranged^9^	Columnar organization^9^	Single cells in lacunae^74^	Single cell, paired, or multiple cells in contiguity^75^
*C. Mechanics and permeability*				
Primary mechanical function	Resist fluid flow in and out of the disc and maintain a uniform stress distribution across the IVD,^86^, ^223^, ^224^ and prevent the disc from herniating or bulging^9^, ^223^, ^224^	Distribute load during joint movement and provide lubricated (low friction) movement^37^	Withstand compressive loads to the IVD and maintain the BEP–CEP interface through fluid pressure^1^	Confine the NP laterally,^7^ and anchor for the IVD to the VB.^48^
Primary permeability function	Main gateway of nutrients and waste into and out of the disc and waste,^6^, ^10^, ^15^, ^50^, ^52^, ^54^ prevent loss of large proteoglycan molecules from the disc^223^	N/A	N/A	Secondary gateway of nutrients and waste into and out of the disc^10^, ^15^, ^50^
Permeability	1.27 × 10^−16^ and 1.66 × 10^−14^ m^4^/Ns^4^, ^10^, ^53^	(0.76 ± 0.42) × 10^−14^ m^4^/Ns^61^	0.67 ± 0.09 × 10^−15^ m^4^/Ns^87^	0.23 ± 0.19 × 10^−15^ m^4^/Ns^87^
Tensile modulus	0.5–21.8 MPa^60^	1–30 MPa^89^	1–1.66 MPa^90^	2.56–12.29 MPa^90^
Bone interaction	Parallel collagen fibers that make it weak and susceptible to detachment^48^	Anchored by perpendicular collagen fibers making them strongly attached^48^	N/A	Outer AF extends to the VB anchoring the disc to the vertebral rim^48^

* Multiple imaging modalities are relevant in distinguishing these structures, expecially when degenerative features are present, e.g. CT in CEP sclerosis.

### 1.2 Developmental biology of the CEP

The CEP, AF, and vertebral bodies develop from the mesoderm, specifically from the sclerotome.^23^ AC is also derived from the mesoderm (Table 1).^24^, ^25^ In contrast, the NP develops from the notochord.^26^ The mesoderm (paraxial) undergoes somitogenesis promoted by precise and cyclic temporal and spatial regulation of Notch and Wnt, and fibroblast growth factor (FGF) signaling pathways, respectively.^27^ The Sonic Hedgehog (Shh) spatiotemporal regulation led by the notochord further differentiates somite to sclerotome and simultaneously promotes spine segmentation.^28^ After sclerotome segmentation, cells proliferate, condense, and undergo chondrogenesis to form the vertebral bone (through the endochondral bone process), the AF, and CEP. This complex regulation is governed by the coordinated action of Shh, SOX5/6/9, Pax1/9, and bone morphogenetic protein (BMP) pathways.^23^

At birth, the human CEP is thicker and takes up approximately half of the intervertebral space, which is reduced to about 5% by adulthood.^26^ Additionally, blood vessels are present at infancy, but are replaced over time by cartilaginous ECM and almost disappear by skeletal maturity.^26^, ^29^, ^30^ In humans, the CEP acts as a growth plate for the vertebrae, but this is lost after teenage years, so that only a thin layer of hyaline cartilage remains.^26^, ^29^ This is different in many animals such as sheep or bovine, in which the growth plate has been shown to persist into adulthood, and is separated from the CEP by the BEP.^31^ The shape of the lumbar (L4–L5) CEP also changes with age, starting with a biconvex shape at infancy, but evolves to a concave shape beginning around the age of 2 or 3 years when children start to walk.^32^, ^33^, ^34^, ^35^ Weight bearing and movement have been shown to influence the shape of the vertebrae and IVDs.^35^ The CEP is vascularized during fetal development; however, by the age of 10 there is a substantial decrease in blood vessels, which are lost by adulthood.^36^ Similarly, blood vessels have been shown to be present in the outer third of the AF up until the age of two, but decrease by age 30 unless there is damage that allows for revascularization.^36^ In contrast, AC^13^, ^37^ and the NP^38^ are avascular regardless of the stage of development.^39^ Throughout the 20s and after adulthood, calcification is observed, often in focal points in the CEP. These calcified sections can drive revascularization and bone formation which occurs following activation of matrix metalloproteinases (MMPs) which degrade the ECM.^29^, ^30^ Furthermore, oxidative stress has been shown to induce CEP calcification through the p38/extracellular signal‐regulated kinase (ERK)/p6 pathway,^40^ which acts in conjunction with mitogen‐activated protein kinase (MAPK) stimulation. This pathway is also involved in cartilage calcification in osteoarthritis,^41^ and is implicated in embryonical endochondral ossification in coordination with the transforming growth factor beta (TGF‐β) and BMP families.^40^, ^42^ Similarly, AF and CEP have both been demonstrated to be the source of pathological fibrocartilage in the NP.^43^ Thus, the ability of CEP to move toward bone phenotype (calcification) and to equivalent AF cell fate (fibrocartilage) suggests that CEP cells maintain developmental‐like plasticity, and consistent tissue homeostasis to maintain their healthy phenotype. Similarly, this de‐differentiation capacity has been shown in articular cartilage (AC), where significant differential expression in the ERK and BMP pathway genes is observed.^44^

## 2 HEALTHY CEP

### 2.1 Structure and composition of the CEP

Throughout life, the composition and anatomy of the CEP and BEP continuously change. During early life, ossification of the VB occurs. While the vertebra‐sided part of the endplates becomes ossified forming the BEP in young adults, the disc‐sided part remains cartilaginous forming the CEP.^30^ The BEP is a layer of porous, coalesced trabecular bone containing pockets of vascularized bone marrow enabling the two‐way transport of nutrients and cellular metabolic products.^45^ In adults, the structure is avascular but has a base that contains a dense network of capillaries formed by terminal branches of metaphyseal and nutrient arteries.^46^ The thicker peripheral section of the BEP forms a junction between the CEP and AF with the vertebral body.^47^

The structural integration of the CEP into the BEP, AF, and NP varies.^1^ At the bone interface there is minor integration, and the bone–cartilage junction is seen as a straight line with no gaps with collagen fibers of the CEP aligned parallel to the bone.^1^, ^48^, ^49^ This is different than AC–bone interfaces, where the collagen fibers of the cartilage are perpendicular to the bone, anchoring the tissue types together.^48^ However, in a healthy disc, the fluid pressure of the NP maintains the CEP and the BEP pressed together and thus, under normal loading (i.e., compression), the limited integration of the CEP and BEP is enough to maintain the BEP–CEP interface.^1^, ^48^ Also at the BEP, there are capillaries that penetrate the pores of the subchondral bone and terminate by looping before the CEP junction.^50^, ^51^ The capillaries are denser at the center of the vertebral endplates above the NP, which is where the IVD is thickest.^52^ As the mature IVD is considered as avascular, the nutrient and waste exchange occur by diffusion from these capillaries.^53^ Although diffusion can occur through the outer AF, the CEP is considered as the main gateway for nutrients into the disc and waste out of it.^6^, ^10^, ^15^, ^50^, ^52^, ^54^ The diffusion distance between the CEP and the cells in the center of the NP can reach 8 mm,^14^ which provides a shorter route for diffusion than through the AF.

**FIGURE 1:**
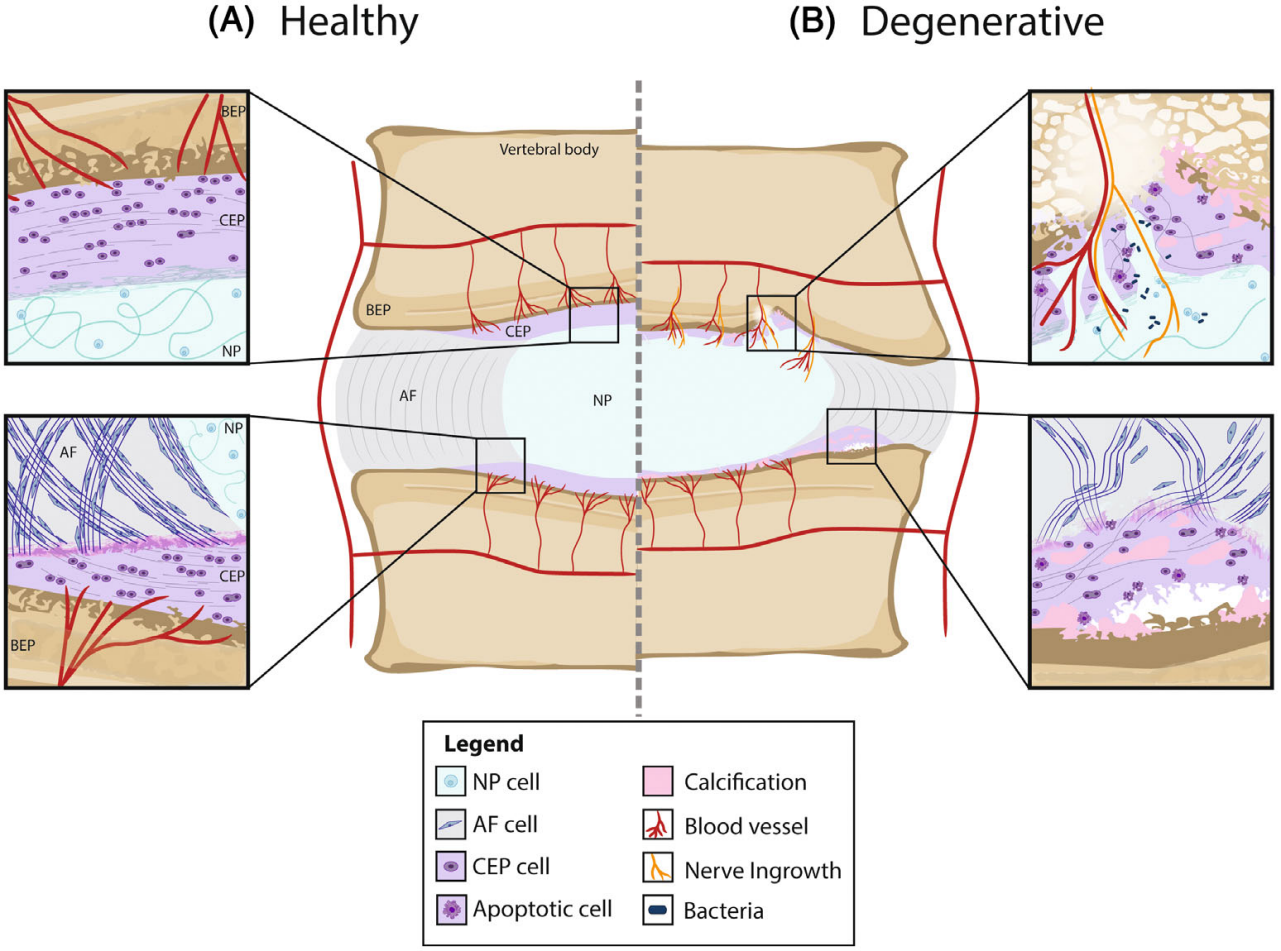
(A) *Healthy CEP*. In the healthy CEP, the collagen fibers of the AF continue into the CEP parallel to the bone (bottom). The collagen fibers of the NP penetrate at least partially into the CEP (top). The BEP–CEP junction is seen as a straight line with no gaps. CEP chondrocytes are rounded, and slightly elongated in the direction of the collagen fibers. The healthy CEP is avascular but has a base that contains a dense network of capillaries formed by terminal branches of metaphyseal and nutrient arteries. (B) *Degenerative CEP*. A degenerated CEP shows loss of thickness, fibrosis, calcification, and apoptotic cells. Fissures allow for blood vessels and nerve ingrowth as well as bacteria entering the NP (top). The adjacent BEP can show sclerosis and signs of MC (top). Avulsions of the CEP from the BEP can also occur (bottom). Integration between the CEP and the NP or AF can also become weaker. These degenerative changes can be identified histologically.^74^ Note that details regarding the other IVD tissues are not included in the image.

In addition, collagen fibers from the outer AF extend into adjacent vertebrae and serve as anchor for the IVD to the rim of the vertebral bone.^48^ The outer AF connects directly into the bone while the inner AF and NP connect to the CEP.^1^, ^48^, ^55^ The integration at the inner AF, however, is more complex as the collagen fibers of the AF lamellae are continuous with those of the CEP (Figure 1).^49^, ^56^ Additionally, SEM analysis of ovine discs has shown that the collagen fibers of the AF branch, which strengthens the annulus–EP anchorage by increasing the interface area over which shear forces are distributed.^57^ These fibers also intertwine and sometimes merge with the fibrils of the CEP. The strength of this connection is essential for the CEP ability to resist tensile loading.^1^, ^49^ Collagen fibers in the CEP differ from both the NP and AF.^58^ The collagen network of the CEP is denser than that of the NP; and tends to be arranged mainly parallel to the vertebrae, although not as highly oriented as in the AF.^9^, ^58^ Additionally, the collagen fibers at the inner CEP are stronger and more interconnected than those at the outer CEP, which could play a role in the anisotropic flow resistance of the CEP.^59^ The highly convoluted collagen fibers within the NP penetrate at least partially into the CEP, providing the resistance to tensile force at the NP‐CEP interface.^7^, ^8^, ^9^

Interestingly, the caudal CEP is usually thinner than cranial one^9^ and both are rich in type II collagen, proteoglycans, and water.^9^ Measurements of cadaveric human lumbar CEPs properties found that collagen content was 681 ± 171 μg/mg dry weight,^4^ or within a range of 32.9%–88.69% dry weight.^60^ In comparison, the collagen content of AC has a smaller range of 60%–70%^61^ while that of the NP was around 20%.^62^ Collagen content of the AF was around 70%^62^ and has a specific alternating aligned orientation of an average ±30°.^63^ The average sulfated glycosaminoglycan (GAG) content was 103 ± 31 μg/mg dry weight,^4^ or within a range of 4.37% and 18.48% dry weight.^60^ The GAG content is similar to that of AC, which ranges between 5% and 15%,^61^ and that of the AF which is 10%.^62^ The NP GAG content, on the other hand, is much higher, ranging between 30% and 50%.^62^ CEP hydration was 1.585–1.666 mg water/mg dry weight, and CEP porosity was 0.648 ± 0.069.^4^ The water content of the CEP, which ranges between 22.1% and 62.4%,^9^, ^60^ is much lower than that of AC, at 70.7%,^64^ the NP, at 70%–90%, and the AF, at 50%–70%.^62^ Chondrocytes are distributed throughout the CEP and are responsible for maintaining the ECM and, thus, providing stability to the tissue.^2^ Macroscopically, CEP chondrocytes are typically rounded, although slightly elongated in the direction of the collagen fibers, more similar to the chondrocytes of the mid or superficial zone of AC than to the chondrocytes of the deep zone of AC.^24^, ^65^ The cell density of the CEP is ~15 × 10^6^ cells/mL with the highest density closest to the vertebral bone. Although similar to the cell density of AC (1.4–1.5 × 10^7^ cells/mL)^66^ this is nearly four times the cell density of the NP (4 × 10^6^ cells/mL) and two times that of the AF (9 × 10^6^ cells/mL).^67^ Recent single‐cell analysis of human IVD cells have demonstrated that there are different IVD cell clusters non‐randomly distributed in the AF, NP, and CEP.^68^ The interactions between these IVD tissues at a mechanistic level are essential to understand the pivotal role of CEP chondrocytes in a healthy CEP structure. The main components of the pericellular matrix around the CEP chondrocytes are hyaluronan, proteoglycans, and type VI collagen,^69^ while interstitial collagen is mainly composed of type II collagen^70^ and proteoglycans, where the type chain, length, and quantity of glycosaminoglycans (GAGs) determine the water content.^71^ Type X collagen, a calcium‐binding collagen which is a key marker of hypertrophy, increases with age and is associated with increased calcification of the CEP.^72^ Additionally, depletion of one of the c*ollagen type II* gene (COL2) alleles has been shown to also promote calcification of the CEP in mice.^73^ Unlike AC, the CEP has a randomly arranged pericellular matrix that does not follow a columnar organization.^9^ In comparison, the NP has single cells in lacunae,^74^ and the AF has single cell, paired, or multiple cells in contiguity.^75^ In addition, the CEP is less hydrated and has lower GAG content than AC,^9^ which is why it should be considered a different and unique tissue. Negatively charged proteoglycans represent approximately 15% of the dry weight of CEP tissue.^4^ CEP cells exhibit an elongated morphology aligned to the collagen‐rich ECM and are arranged parallel to the VB, similar to superficial zone chondrocytes of AC, while deep cartilage AC cells present a round morphology and are arranged perpendicular to the adjacent bone.^24^, ^65^ In contrast, the NP has fibrochondrocyte‐like cells.^76^ The cells of the AF differ between the inner AF, where cells are rounded and chondrocyte‐like, and the outer AF, where cells are elongated, fusiform, and fibroblast‐like.^77^ Furthermore, AC cells present a decreased expression of ECM genes (*ACAN*, *COL1A1*, *COL2A1* genes) relative to CEP cell expression.^65^ Other possible gene markers suggested for the CEP include *ERK*, *BMP*.^51^ Genetic markers of AC have been proposed as *GDF10*, *CYTL1*, *IBSP*, *FBLN1*,^78^ *ACAN*, and *PTN* ^79^ while NP markers are considered as *PAX1*, *FOXF1*, *HBB*, *CA12*, *OVOS2*,^78^ *KRT19*,^80^ *ACAN*, *VCAN*, *TNMD*, *BASP1*, *TNFAIP6*, *FOXF1*, *FOXF2*, and *AQP1*.^79^ Genetic markers of the AF have been indicated as *COL1*, *VCAN*, *PTN*, *TNMD*, *BASP1*, *TNFAIP6*, *FOXF1*, *FOXF2*, and *AQP1*.^79^

### 2.2 Mechanics and permeability within the CEP

#### 2.2.1 Effects of pressurization on CEP mechanics and permeability

Mechanical forces are crucial in maintaining cartilage homeostasis.^81^ Chondrocytes respond to the mechanical environment, which contributes to the regulation of cell metabolism. As with other cartilage tissues, mechanical loading, such as compressive, tensile, and shear forces as well as pressure from fluid flow, is essential for the function of the CEP.^82^ While overloading of the disc will result in vertebral endplate fractures or another injury, lack of mechanical stimuli will also impair disc homeostasis.^83^ As it sits above and below the NP, the CEP acts as a mechanical barrier that adds resistance to the flow of fluid from the IVD to the VB, allowing for the pressurization of interstitial fluid in response to compression while preventing the disc from bulging into the adjacent VB.^58^, ^84^, ^85^ This fluid pressurization helps to maintain a uniform stress distribution across the IVD.^86^ Thus, the permeability of the CEP is essential in maintaining the intradiscal pressure. In contrast, AC mechanically distributes load during joint movement and provides lubricated (low friction) movement.^37^ However, it does not play an important role in permeability.

The reported permeability of cartilaginous tissue varies significantly. AC has been reported to have a permeability of 0.76 ± 0.42 × 10^−14^ m^4^/Ns^61^ while the NP has a permeability of 0.67 ± 0.09 × 10^−15^ m^4^/Ns,^87^ and the AF has a permeability of 0.23 ± 0.19 × 10^−15^ m^4^/Ns.^87^ The permeability of the CEP ranges between 1.27 × 10^−16^ and 1.66 × 10^−14^ m^4^/Ns,^4^, ^10^, ^53^ depending on the CEP location in the IVD, the animal model, and the region of the sample within a CEP. Rodriguez et al. also reported a permeability of 1.19 × 10^−10^ m^4^/Ns in human CEPs, and explained the significantly lower measured permeability as due to inhomogeneities and focal cartilage lesions common in degenerated human samples.^52^ Despite the considerable variation in the reported CEP permeability, it is still at least an order of magnitude less than that of the BEP (~2.21 × 10^−9^ m^4^/Ns).^4^, ^51^, ^52^, ^88^ However, a more accurate representation would consider the CEP permeability as a gradient which exponentially increases from the NP toward the BEP according to proteoglycan content.^10^ Early studies using disulfine blue dye also demonstrated higher permeability across the CEP at the center than across the lateral regions.^15^ However, despite this, the central region of the NP experiences low nutrition and high lactate concentrations due to the large diffusion distances across discs, particularly in the lumbar region.^54^ Further, there are differences in permeabilities in the CEPs of the same IVD, in which the cranial CEP is significantly more permeable than the caudal one^9^ possibly due to the differences in loading experience.

#### 2.2.2 Effects of biochemical composition on CEP mechanics and permeability

The biochemical composition is fundamental in determining the material properties of the CEP, and therefore the permeability and response to mechanical loading.^60^, ^85^ It has been proposed that there is an optimal range of biochemical composition that balances both the biomechanical and nutritional demands of the CEP.^60^ To this extent, the CEP must be stiff enough to hold the disc together but porous enough to allow for solute transport. Therefore, the CEP tensile properties have been found to be inversely related to the transport properties.^60^ Additionally, within the bovine CEP, the biochemical composition, and therefore the biomechanical properties, were found to vary in the central region located next to the NP, compared with the lateral CEP, which is stiffer and thus could withstand a more significant portion of loading.^85^

The tensile stresses within the CEP occur through Poisson's effects when the CEP is pressed against the BEP by the NP pressure, and/or through direct peripheral pulling by the inner AF fibers that blend with the CEP. At the inner AF and NP, the collagen is not as highly oriented as the outer AF, and therefore does not exert high tensile forces on the CEP,^1^ although the central CEP will experience transverse shear and some tensile stress when the NP bulges laterally during compression.^49^ The tensile modulus of the CEP was found to be 5.9 ± 5.7 MPa, ranging from 0.5 to 21.8 MPa.^60^ In comparison, the tensile modulus of AC ranges from 1 to 30 MPa,^89^ NP from 1 to 1.66 MPa, and AF from 2.56 to 12.29.^90^ The tensile modulus of the CEP positively correlates to the collagen content; however, water and GAG content have been shown to have minimal effects.^60^ In contrast, water content is expected to play a more prominent role than GAG content in the extrinsic viscoelastic, or poro‐elastic, properties, that is, aggregate modulus and hydraulic permeability, and diffusion of the CEP, alongside other cartilage tissues including the NP, AF, and AC.^9^, ^58^, ^85^ Yet, GAG content is considered more important in osmotic properties, rather than elastic properties in the CEP.^9^ In particular, multiphysics models showed that this characteristic might provide the GAG with an important role in the effective control of the fluxes of fluid between the BEP and the NP, by the CEP.^10^ Interestingly, GAG quantity has been shown to not correlate with water content,^9^ suggesting that the type and quality of the GAGs are more important than the quantity.

**FIGURE 2:**
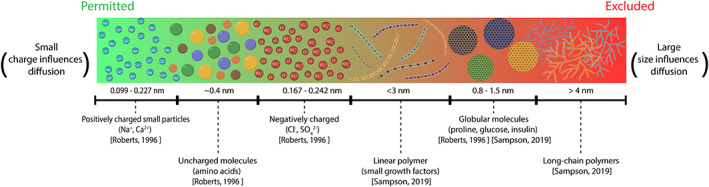
Healthy CEP diffusivity to different types of solutes based on their charge for small molecules and/or on their size and shape for large molecules. The molecules and diffusivities are based on Roberts et al.^6^ and Sampson et al.^4^

CEP transport properties depend on the porosity and collagen, GAG, and water content of the CEP matrix,^6^, ^51^ while solute transport into and out of the IVD depends on solute size,^50^ shape, weight,^6^ and charge (Figure 2). For small molecules, net charge is the determining factor of diffusivity through the CEP.^5^ The charge of the molecules are important due to the Donnan osmosis effect, in which small positive ions from the interstitial fluid migrate into proteoglycan‐rich tissues.^91^ Water enters the tissue to equilibrate the chemical potential, and the tissue swells as much as allowed by the collagen network and surrounding tissue constraints. Thus, electrical charge of small particles impacts the diffusivity.^92^, ^93^ Further, multiphysics models suggest that proteoglycan content has a greater effect than collagen content on the macroscopic hydraulic permeability of the CEP.^10^ Nevertheless, when biopsies or cores of CEP are used it is challenging to control potential GAG loss and tissue swelling in altered osmotic environments in vitro compared with those seen in vivo. Thus, future studies where GAG release into media is prevented and osmotic pressure is controlled are essential to further the understanding of CEP permeability.^94^

Conversely, for large electrically uncharged molecules, size is the determining factor of diffusivity as the space between GAG chains of aggrecan is only ~3–4 nm.^6^ Due to their ability to bend, linear polymers have a relatively high diffusivity compared with spherical molecules of the same molecular weight.^4^ However, long‐chain polymers have a relatively low diffusivity compared with globular molecules of the same, or even higher, molecular weight. For example, starch, a globular 10 kDa molecule, diffuses more than polyethylene glycol (PEG), a long‐chain 4 kDa polymer.^6^

Additionally, dynamic loading influences convective solute transport of large solutes, particularly in less porous CEPs.^4^, ^58^, ^95^, ^96^ However, dynamic compression has been shown to have a minimal effect on small molecule, such as glucose or lactate, transport as this acts primarily through diffusion.^4^, ^94^ In contrast, static compression can decrease diffusivity and inhibit nutrient transport because the tissue gets compacted, and thus porosity decreases.^58^, ^82^

#### 2.2.3 In silico investigation of CEP multiphysics

In silico simulations, such as finite element (FE) and computational fluid dynamics (CFD) analyses, have been widely used to determine the multiphysics mechanisms involved in biological tissues such as the ones of the IVD, in response to external mechanical loads. Models are useful in understanding the particularity of CEP mechanics. However, despite its importance, the CEP is often disregarded in in silico simulations, simplified to a boundary condition of the IVD,^97^ or given homogenized properties.^98^ Since experimental data on the CEP is limited, equations and material properties determined for the IVD or AC are often used to represent the CEP.^10^ Osmo‐poroelastic models have been demonstrated to be generic enough to use for cartilaginous tissues made up of proteoglycans and collagen fibers, however, CEP‐specific data should be used when possible.

Nevertheless, models have given insight into how the IVD gains water during 8 h of rest at night much faster than it loses water during 16 h of activity during the day. The compression of the CEP against the BEP is considered to close the porosity of the CEP and limit water *out‐flow* when the IVD is compressed, while opening the porosity and favoring fluid *in‐flow* into the IVD when unloaded.^99^ Coined as the “intervertebral disc valve theory,” this mechanism might explain functional anisotropic, or direction‐dependent, flow resistance mechanism in the CEP^100^ and has been backed up by experimental measurements^100^ and independent permeability measurements.^53^ Further, in silico simulations used composition‐dependent CEP modeling and incorporated cranio‐caudal gradients of compositions measured in a healthy human disc to support the theory.^2^ These simulations found that CEP porosity changes additionally induced by the composition gradients reinforced the resistance to the mass flow of water that reaches the BEP when the NP is pressurized.^10^ However, there are contradictory findings on whether the favored direction of flow at the CEP is *in‐flow* or *out‐flow*.^94^, ^101^

## 3 IMPORTANCE OF CEP IN IVD DEGENERATION

The integrity of the CEP and BEPs are key to the homeostasis of the motion segment as they form an interface to exchange nutrients and metabolites from the IVD to the external circulation and play an essential role in the mechanical stability of the motion segment. Thus, pathological processes that occur to the CEP and BEP can alter the mechanical and nutritional environment of the IVD, triggering degeneration. Although most studies have focused on changes to the BEP with degeneration, which are visible on MRI images, these are likely associated with CEP changes as well. Indeed, cells derived from CEP adjacent to degenerated discs have very similar properties (morphology, immune phenotyping, proliferation, and gene expression) to bone marrow mesenchymal cells from the same patients.^21^ BEP defects have been associated strongly and independently with IVD degeneration,^102^ where they have been hypothesized to be an initiating factor to degeneration of the disc.^33^, ^103^, ^104^, ^105^

### 3.1 Imaging endplate defects

**FIGURE 3:**
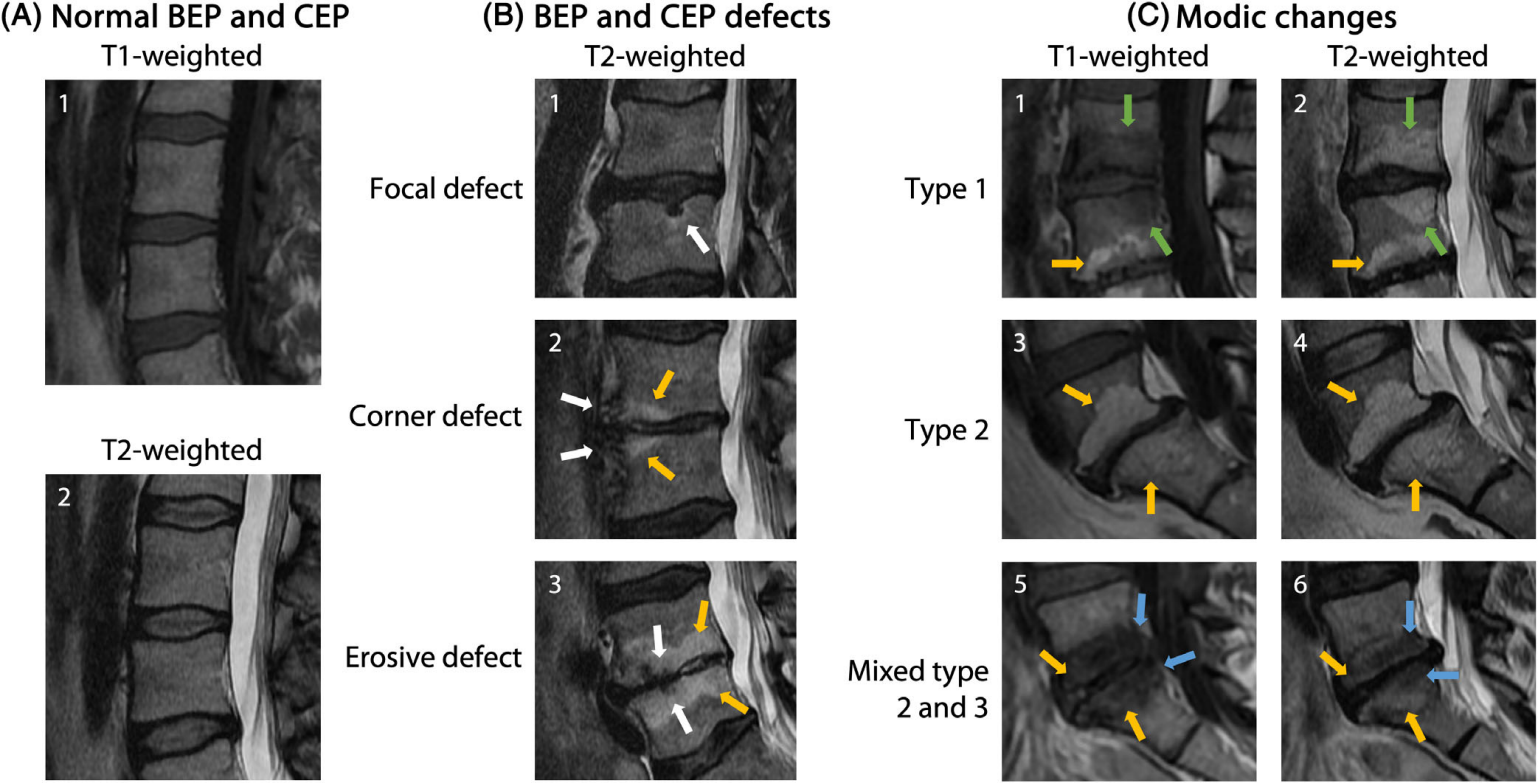
Examples of CEP and BEP appearance in standard of care MRI from 47‐year‐old participants in the Northern Finland Birth Cohort 1966. (A.1–A.2) Characteristic appearance of healthy CEP and BEP on T1‐ and T2‐weighted MRI. (B.1) Schmorl's node as an example of a focal defect at the L3–L4 IVD caudal BEP (white arrow); (B.2) corner defects at the anterior edges of the L4–L5 IVD cranial and caudal BEP (white arrows) with accompanying type 2 MC (orange arrows); (B.3) erosive defects at the L4–L5 IVD cranial and caudal BEP (white arrows) with accompanying type 2 MC (orange arrows); (C.1–C.2) type 1 MC extending from the L3–L4 IVD cranial and caudal BEP (green arrows) with type 2 MC also visible in the L4 vertebral body (orange arrows); (C.3–C.4) type 2 MC extending from the L5–S1 IVD cranial and caudal BEP (orange arrows); (C.5–C.6) type 2 and type 3 MC (orange and blue arrows, respectively) extending from the L5–S1 IVD cranial and caudal BEP.

BEP defects include several key recognizable features, which are normally identified on magnetic resonance imaging (MRI) images (Figure 3). Computed tomography (CT) has been used to identify the presence of endplate sclerosis in MC but is not usually suitable for clinical diagnosis or epidemiological studies.^106^ Generally, the term “endplate changes” in literature describing clinical T1‐ and T2‐weighted MRI features refers to changes seen in the bone marrow adjacent to the CEP, typically referred to as Modic changes (MC). Atypical changes that affect the endplate which are detectable from MRIs can be classified into three categories: focal, corner, and erosive.^107^ Focal changes, in which Schmorl's nodes are included, are defined as local hollow regions on the endplate with NP protrusion into the subchondral bone; while corner defects are changes in anterior or posterior end of the BEP with the compromise of the vertebral trabeculae. Finally erosive defects are characterized by an irregular extensive alteration of the endplate on T2‐weighted images.^107^ However, some features, such as endplate changes in the upper lumbar spine may have a developmental rather than degenerative origin.^108^ Moreover, in a large population‐based study BEP damage was strictly associated with MC, rather than other endplate defects.^109^

Modic changes, although by definition connected to the CEP, are at best an indirect reflection of CEP status and do not correlate well with histology.^110^, ^111^ The CEP itself is not clearly visible with conventional MRI sequences. Cartilage has short T2 values, so the CEP signal is not detected or is very hypointense. Additionally, its size ranges from 0.1 to 1.6 mm,^1^ which is close to the typical pixel size of a sagittal T2‐weighted MRI of 0.5 mm^2^. This poses a significant challenge for identifying the role, if any, of the CEP in LBP through imaging.^112^ In contrast, AC is distinguishable from adjacent soft tissue and bone.^113^ In healthy discs, the position of the CEP can be inferred as a line of hypo‐intensity between the hyper‐intense NP and the VB on T2‐weighted MRI but it is impossible to distinguish this from the BEP (see fig. in Law et al. (2013)^114^). However, this feature is obscured when the AF is hypointense, which is characteristic of the early stages of degeneration.

The terms “Schmorl's nodes” and “endplate damage/disruption” are often used to describe specific MRI features at the boundary between the IVD and the VB seen on MRI, making them more specific to the CEP. However, the multiple proposed classification schemes for these imaging features reflect a poorly defined phenotype.^32^ Considering the limitations of T1‐ and T2‐weighted sequences and CT for analyzing CEP features, alternative quantitative MRI approaches have been implemented.^115^ Ultrashort time to echo (UTE) MRI is the most widely reported as an effective means of visualizing the CEP and can show in vivo the delineation of the BEP and CEP.^114^, ^116^ Outside of the clinical setting, both CT and MRI are widely used in ex vivo animal or human studies. For example, MRI has been used in ex vivo human cadaveric spine segments to characterize the structure of the CEP in detail.^117^ microcomputed tomography (μCT) has also been used in such settings to confirm compositional characteristics of the CEP seen in MRI such as sclerosis,^118^ or for detailed morphological and biochemical characterization using contrast enhanced μCT.^119^ These ex vivo imaging approaches are needed to better understand the role of early CEP changes in the pathogenesis of IVD degeneration, while clinically applicable sequences and accompanying analyses capable of detecting early CEP changes are needed for identifying at‐risk patients and early intervention targets.

### 3.2 Significance in low back pain

The evidence for innervation of the vertebral bone marrow extending to the endosteal surface gives a biological basis for the endplate as a source of nociception in vertebrogenic pain.^120^ Areas of vertebral endplate damage may trigger neoinnervation,^120^, ^121^, ^122^ with concomitant bone marrow pathologies (MC) also shown to be innervated.^120^, ^123^, ^124^, ^125^ While these sensory fibers generally terminate in or near the endosteal surface of the BEP, the CEP has also been shown to have small vascular spaces containing nerve fibers in some cases^121^ and the CEP specifically can be accessed by nerves and blood vessels in the case of damage.^126^

Endplate sensory fibers may be activated mechanically and chemically in the case of endplate damage. Disc/vertebra crosstalk as a consequence of the breakdown of the barrier provided by the CEP can contribute to nerve irritation with exposure to proinflammatory and neurogenic factors and the by‐products of NP anaerobic metabolism such as lactic acid.^18^ Additionally, damage to the endplate alters the distribution of IVD stress and the response to spinal loading,^18^, ^45^ contributing to local mechanical nerve activation. Associated changes to the paraspinal muscle quality may further interfere with segmental biomechanics and play an aggravating role in endplate nociception.^127^

Although established as a plausible source of nociception, as with other spine image phenotypes, the observed relationship between visible defects of the vertebral endplate and the experience of pain is unclear. Multiple grading schemes for qualitative grading have been put forward,^128^, ^129^ but no consensus exists for endplate MRI image phenotype nomenclature,^32^ which makes it more challenging to interpret and aggregate pain association study results. There is also a limitation in the ability to specifically detect CEP changes using clinical diagnostic tools (e.g., standard T1‐ and T2‐weighted MRI sequences), and these cannot distinguish changes specifically associated with neoinnervation. Alternative sequences such as UTE^115^ are not widely used in the clinical setting, and few methods for quantitative image analysis of the endplate have been tested.^112^, ^130^

### 3.3 Modic changes: Definition, prevalence, natural course, and pain association

MC are MRI signal intensity changes of the vertebral bone marrow around a degenerated IVD^104^, ^131^ and independently associate with chronic low back pain (LBP).^132^, ^133^, ^134^, ^135^, ^136^ A meta‐analysis showed that MC prevalence in LBP patients is about seven times higher than in the non‐clinical population (43% vs. 6%). MC occur predominantly in the lower lumbar spine.^137^ There are three interconvertible types of MC depending on their appearance on T1‐ and T2‐weighted MRI. Modic type 1 changes (MC1) (Figure 3) are hypointense on T1‐weighted images and hyperintense on T2‐weighted images and represent edema, fibrovascular granulation tissue, infiltration of immune cells, and expansion of profibrotic stromal cells.^131^, ^138^, ^139^, ^140^ Modic type 2 changes (MC2) are hyperintense on T1‐ and T2‐weighted images and represent fatty marrow conversion with presence of fibrotic tissue.^131^, ^140^ Modic type 3 changes (MC3) are hypointense on T1‐weighted and T2‐weighted images and are sclerotic changes.^131^, ^141^ The reported prevalence for MC1, MC2, and mixed type (MC1/2, MC2/3) are highly variable. Median prevalence is around 15%–20% for MC1, 25%–65% for MC2, and <5% for MC3.^132^, ^142^ Mixed type MC1/2 are also frequent (15%–20%), whereas mixed type MC2/3 are rare. The prevalence generally increases with age and peaks in the 60s.^143^, ^144^ MC can inter‐convert over time.^131^, ^145^, ^146^, ^147^, ^148^ MC1 are the least stable, where within 4 years, most MC1 either convert to MC2, increase in size, or resolve. MC2 are more stable, while MC3 are a terminal stage. Smaller MC lesions are more likely to resolve than larger lesions.^145^

The association of chronic LBP with MC has been extensively studied and reviewed..^149^ About half of the studies report a significant association with odds ratio ranging from 1.53 (95% CI: 1.02–2.29) to 83.10 (95% CI: 4.85–1424) Only one study reported a significant negative association with MC2 with an odds ratio of −3.2 (CI: −5.39 to −0.1). The association of MC with discography concordant pain has a specificity of >94% in five of six studies with a OR of 4.01 (1.52–10.61) in a meta‐analysis.^18^, ^149^ Larger lesions had a stronger association with discography‐concordant pain.^150^ Overall, these data show an association of MC with LBP, in particular of MC1.

Increased innervation of the CEP in MC1 and MC2 is believed to cause increased pain sensitization at MC levels and is often referred to as vertebrogenic LBP.^120^, ^124^ Low back vertebral endplate pain (DM54.51) has recently been added to the International Classification of Diseases (ICD‐11) as a subclassification of patients with LBP and MCs.

### 3.4 Association of BEP changes with CEP changes and MC

Despite the importance of CEP and BEP in the onset of spinal pathologies, the relationship between CEP and BEP is poorly understood due to the difficulty of evaluating the CEP with imaging techniques. A study on cadaveric lumbar spines using ultrashort time‐to‐echo MRI was used to enable investigation of CEP morphology within IVDs with BEP lesions, demonstrating abnormalities of the CEP were statistically associated with BEP lesions.^151^ After needle induction of IVD degeneration in a rabbit animal model, the CEP progressively thickened and showed increased collagen accumulation.^152^ Along with changes in cartilaginous tissue, the bone interface was modified, specifically an increase in bone volume fraction. These findings suggest that CEP could have a role in the development of BEP lesions or vice versa. It has been shown that poor CEP composition can affect disc health with and without defects in the VB.^153^ However, a precise understanding of the sequelae of changes in the CEP and BEP is still missing. There is comparably little data available about the integrity and damage of the CEP in MC. Fields et al.^120^ showed that in cadaveric human spines, CEP damage is associated with histological changes consistent with MC. Still, not all specimens with histopathological changes had MC on MRI. In another human cadaveric study, Heggli et al.^125^ showed that CEP and BEP damage are strongly associated with MC2. Supporting evidence for CEP damage in MC stems from studies assessing CEP fragments in surgically removed herniated disc tissue at the MC level. CEP fragments can co‐herniate with disc tissue in cases of avulsion‐type herniations, where the CEP is torn from the BEP. These CEP avulsions were found to associate with MC.^154^, ^155^

### 3.5 Mechanisms of CEP damage in Modic changes

While damage to the BEP is believed to be caused mainly through mechanical cues, mechanisms of CEP damage are poorly understood.^156^ In MC, local biological reactions seem to contribute to CEP damage. In an animal model of MC1, immune reactions in the MC1 bone marrow caused damage to the adjacent endplate. This is noteworthy because it demonstrates that MC1 are not just reactive changes to disc degeneration, but that MC1 themselves can cause CEP damage and maintain the cross‐talk of the bone marrow with the adjacent disc.^157^ Recent studies confirm the possibility that activated immune cells in MC1 bone marrow can lead to CEP damage.^158^ Increased lactate dehydrogenase activity and increased concentration of C‐reactive protein and of complement factors in MC bone marrow indicate also a humoral immune response related to local tissue damage.^125^, ^159^ On a cellular level, CEP cells at MC levels express more tumor necrosis factor (TNF), a disintegrin and metalloproteinase with thrombospondin motifs‐5 (ADAMTS‐5), macrophage migration inhibitory factor (MIF), and its receptor CD74.^124^, ^160^, ^161^ TNF upregulates MIF in CEP cells, and MIF upregulates the secretion of proinflammatory cytokines by CEP cells, through an autocrine mechanism involving CD74. The existence of this positive inflammatory feedback loop suggests that the CEP has the capability to escalate inflammation in MC independently from the disc and the bone marrow. Together, these data evidence that CEP damage in MC is not a pure mechanical mechanism but that the local inflammatory processes in the bone marrow and of the CEP itself can lead to progressive CEP damage.

Occult disc infection, mainly with the *Cutibacterium acnes* (Gilchrist 1900) and other coagulase‐negative staphylococci are discussed as a potential etiology of MC1, at least in a subset of patients.^144^, ^162^ This is based on reports in which disc tissue from microdiscectomy of herniated discs was analyzed for the presence of *C. acnes*.^144^, ^163^, ^164^, ^165^ *C. acnes* has been isolated from discs adjacent to MC1 and the presence of this bacteria was predictive for the development of new MC1 after 1 year. *C. acnes* is assumed to migrate to structurally damaged discs through hematogenous spread from a distant infection or from the skin and other epithelial surfaces through the blood after innocuous lesions, for example, tooth brushing.^166^, ^167^ Disc herniation and endplate damage, both structural damages present in MC, can represent disc damage which allow bacteria to enter discs. Once in the disc, the low oxygen tension and low pH in the disc favor the proliferation of *C. acnes*. Furthermore, *C. acnes* is unlikely to colonize the MC1 bone marrow because of too high oxygen tension in the bone marrow. Rat and rabbit models have demonstrated the biological plausibility that *C. acnes* injected into disc can trigger MC1‐like changes.^168^, ^169^, ^170^ For example, injecting a *C. acnes* strain, which had been isolated from a human MC1 disc triggered hallmarks of MC1 within 2 weeks after injection (i.e., MC1‐like MRI changes in the bone marrow, disc degeneration, fibrotic‐inflammatory bone marrow changes, and almost complete resorption of the CEP).^170^

The exact mechanism of how intradiscal *C. acne*s causes CEP resorption is still unclear. It has been shown that disc cells respond to *C. acnes* with the release of proinflammatory and neurotrophic factors through a toll‐like receptor 2 (TLR2)‐dependent pathway.^170^, ^171^, ^172^, ^173^ Stimulation of TLR2 on disc cells also upregulates matrix proteases that can degrade the disc and CEP matrix. Additionally, *C. acnes* secrete different virulence factors such as proteinases, hyaluronidases, lipases, and neuraminidases.^174^, ^175^ Proteinases and hyaluronidases can directly degrade ECM components of the CEP. Lipases hydrolyze triacylglycerides into glycerol and free fatty acids. Free fatty acids, in particular saturated free fatty acids, are highly proinflammatory by signaling through toll‐like receptor 4 (TLR4).^176^, ^177^ Neuraminidases disrupt the ternary complex of TLRs with the membrane components CD24 and SiglecG/10 and abolish the inhibition of TLR signaling by SiglecG/10.^178^ Together, *C. acnes* has the capacity to cause CEP damage, yet the precise mechanism remains unclear.

### 3.6 Role of CEP damage in Modic changes

Damage to the CEP compromises its function as a sieve for cells and macromolecules. In MC, where endplate damages are present, the degenerating disc can cross‐talk with the adjacent bone marrow.^157^ Proinflammatory and pro‐osteoclastic cytokines that are produced at higher rates from MC discs can more easily escape into the adjacent bone marrow.^156^, ^157^, ^179^ Marrow‐sided leukocytes can in turn aggravate degenerative changes in the disc, even without infiltrating the disc.^156^ Consequently, CEP damage in MC facilitates an inflammatory cross‐talk between the disc and the bone marrow that contributes to the rapid degeneration of MC segments.^104^ Furthermore, increased concentrations of cytokines and chemokines produced during disc degeneration^180^, ^181^ could more easily diffuse out of the IVD into the adjacent bone marrow leading to chemotaxis gradients and activation of immune cells.

### 3.7 Microscopic changes to the CEP

**FIGURE 4:**
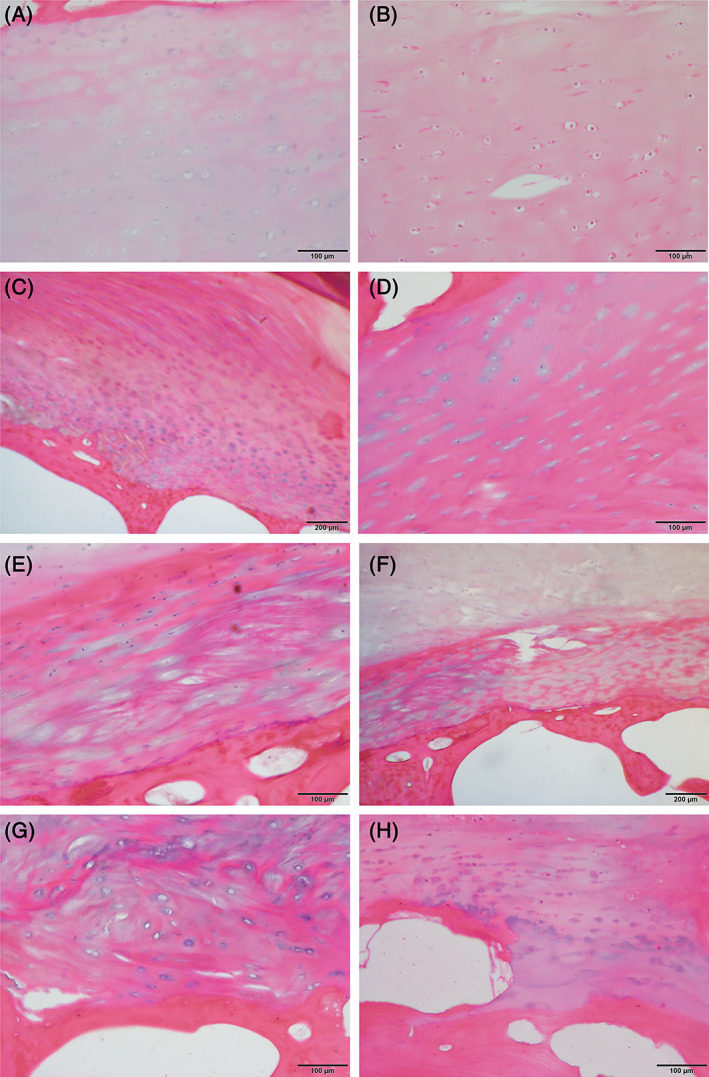
Hematoxylin and Eosin staining of human cartilaginous end plates (CEP) demonstrating key histological features of non‐degenerate and degenerate CEP. (A) Non‐degenerate CEP with BEP top left of image, CEP within region of the NP. (B) CEP within the region of NP demonstrating excellent maintenance of eosin staining. (C) CEP/AF enthesis within a non‐degenerate region, image shows BEP at bottom left and CEP within the middle connecting into AF. (D) CEP within region of AF tissue demonstrating change in matrix organization at bottom of image. (E) Abnormal CEP demonstrating clear disorganization and fibrosis. (F) Abnormal CEP showing fissures and disorganization of the CEP. (G) Abnormal CEP showing disorganization of the extracellular matrix. (H) Abnormal BEP/CEP enthesis with boney evulsion shown. Scale bars as indicated: 100 μm (A, B, D, E, G, and H), 200 μm (C and F).

During IVD degeneration, key structural and cellular changes occur within all areas of the IVD, with key features identified within the NP, AF, CEP, and BEP (Figure 1). While macroscopic structural changes can be identified by imaging techniques such as MRI, these often fail to identify changes to the CEP. Furthermore, cellular and fine ECM changes require microscopic examination to be identified. (Figure 4) During the development of the recently published standardized histopathology scoring system for human IVD degeneration,^74^ each of the regions (NP, AF, CEP, and BEP) were scored independently with equal weighting, demonstrating the recognition of the critical role of the CEP and BEP in IVD degeneration.^74^ Within the CEP the degenerative features identified histologically included scoring for cellularity, lesions and ECM structure.^74^ Cellularity changes seen during degeneration included: abnormal cellular clusters, empty lacunae, extensive neovascularization, and presence of apoptotic, necrotic, and senescent cells.^74^ Changes within ECM can be identified histologically, and include loss of endplate thickness; avulsions from BEP; cracks and fissures; loss of normal matrix staining; fibrosis and calcification.^74^ These visible microscopic changes within the CEP are due to alterations in the biomechanical and cellular regulation of the CEP.

Additionally, through histological analysis Huang et al.^182^ identified physical microdamage in 40% of degenerated CEPs. Further, pain and disability scores were significantly higher in these microdamaged CEPs than in degenerated IVDs without damaged CEPs. Six main patterns of microdamage were used to classify the CEPs, including fissures, vascular mimicry, NP herniation into the CEP, NP herniation and incorporation of bone tissue into the CEP, incorporation of bone tissue into the CEP, and traumatic nodes.^182^

Another methodology used to categorize the endplate is the total endplate score, or “TEPS.”^183^ MRI scans are used to quantify the damage, which was given a score based on diffusion patterns and the presence of endplate breaks or defects, focal thinning, MC, and irregularities or sclerosis. Five visually distinct diffusion patterns were identified through the study as well, that reflected disc marrow contact, focal leakage into the subchondral bone or NP, and pooling of liquid. These diffusion patterns correlated with degeneration level as well as the TEPS. As the TEPS increased, the diffusion pattern increased toward pooling. The TEPS was shown to correlate with Pfirmann's grading regardless of the age and levels of disc. Unfortunately, there was no distinction between CEP and BEP in that study.

### 3.8 Biomechanical regulation of CEP degeneration

There are two main theories about the contribution of mechanical loading to IVD degeneration; the overload theory, which states that excessive mechanical loading damages the IVD over time, and the immobilization theory, which states that low mobility causes the IVD to adapt and leads to tissue weakness and degeneration.^83^ There is evidence that both overloading and immobilization contribute to IVD degeneration, and it is considered that there is a range of mechanical loading in which the disc remains healthy. Outside of this range, the metabolism shifts to catabolism.

The CEP is essential in maintaining the mechanics of the disc. Damage to the CEP barrier alters the hydration and could allow water to escape from the NP under loading, leading to NP decompression and degeneration.^1^ FE simulations of the disc, performed in the 90s, suggested that initial failure always occurred in the endplates, demonstrating their vulnerability within the disc under loading.^184^ Nevertheless, experimental testing of disc failure show that although most failures happen at the CEP–bone interface or disc–CEP interface, some primary failures also occur within the subchondral bone,^48^ which was not predicted by such early FE models.^184^ Additionally, endplate herniation through the CEP is a key feature seen in patients and is the most common type of herniation.^185^, ^186^ However, after endplate damage, additional failures could propagate to the annulus or lead to delamination.^184^ This highlights the importance of CEP integrity; preventing CEP damage early on could also avert damage to the rest of the disc and prevent disc degeneration.

Cyclic compression has been shown to lead to the development of microstructural voids at the CEP‐BEP border.^187^ Microdamage accumulation occurred more often in flexed‐joint postures in comparison to neutral postures. Following the damage, there was also a decrease in type I and II collagen content. Thus, overuse injuries could occur in the CEP and lead to altered biochemical content.

The IVD is most vulnerable to failure under bending, in which stretching puts the disc under tension.^1^, ^48^ Studies have found that in cadaveric thoracic IVD samples under tension, 71% of primary failures occur at the CEP‐BEP interface.^48^ The next most common failure was at the IVD‐CEP interface (21%), while the rest failed within the subchondral bone. When pulled in tension from the vertebral bone on each side, the tensile failure strength of the CEP‐BEP interface is 0.4 MPa,^48^, ^49^ while failure strain, calculated from force–displacement data normalized to the initial specimen height, was found to be 38.5% ± 20.3% with the highest principal strains occurring in the mid‐AF.^48^ The rate of loading also affects the failure; slower rates cause disc pressurization with localized stretching and failure in the AF, while under rapid loading the AF does not have time to stretch and thus pulls the CEP from the bone.^188^ Further, the position of the disc affects the failure location; neutral discs, which would be seen during normal standing, tear at the CEP‐inner AF interface, while flexed discs, which would be seen during bending movements, fail at the outer AF‐endplate interface or in the BEP.^188^, ^189^

The thickness, porosity, and curvature of the CEP also influence the biomechanics.^48^, ^49^ Further, Thompson Grade and bone density have been correlated with failure strength.^48^ Additionally, bone volume fraction (bone volume (BV)/total volume (TV)) has been shown to positively correlate to the failure strength, with stress increased at the higher BV/TV end of the VB.^45^, ^48^, ^190^ In humans, the cranial endplates have a lower BV/TV than caudal endplates, and therefore they tend to fail before the caudal endplate fail.^45^ The microarchitectural features of endplate concavity are also significant predictors of failure strength.^190^ Specifically, when the concavity of the CEP is wider, more voluminous, and less steep, it is capable of tolerating higher loads before failure.^190^ Overall, CEPs are stronger when they are thicker and denser with a higher concave curvature that allows more space for the NP.^48^, ^49^, ^190^, ^191^ Thickness has even been suggested to be used as a clinical risk measure for avulsion‐type herniation.^48^ It should be noted that quadrupeds have a different curvature than humans, as well as additional material properties such as BV/TV, and therefore it is essential to consider the animal model used during mechanical tests.^190^, ^191^

### 3.9 Animal models of the CEP

As human CEP samples are not always available, it is important to consider animal models which can be used to elucidate the properties and functions of the CEP. Interestingly, rats and mice have only a CEP with no BEP.^31^ Rabbits and goats have a very thin CEP (1–3 cell layers) with a larger BEP. Larger animals such as dogs, cows, and sheep have both; however, the CEPs are thinner and the BEPs thicker than those of humans.^20^ Bovine and canine CEPs have been shown to have similar biochemical content to human CEPs, although canine CEPs have been shown to have significantly more sulfated GAG than those of humans.^20^ Further, bovine CEPs have been shown to have more proteoglycans in the outer AF–EP region in comparison to the NP–EP region similar to the human CEP, while canine CEPs show the opposite pattern. Bovine CEP cells are rounded and organized in stacked columns, in contrast to canine cells which have no organization and human CEP cells which are along the collagen fibers parallel to the disc. Thus, the molecular similarities of the bovine and human CEPs make the bovine a more suitable model for investigating mechanics and transport in the CEP.^20^ Additionally, baboon CEPs have been demonstrated to have similar biochemical content, including GAG, water and collagen, as those of humans, and thus could also be a good model to study mechanics with the CEP.^86^ Rabbits have also been validated as a model to investigate initial endplate failure, although rabbit endplates have a higher BV/TV and a steeper, narrower concavity which should be considered when translating results to humans.^190^ Particular care should be taken with diffusivity studies, taking into account the difference in thickness and CEP:BEP ratio compared with the human endplate.^20^ Overall, no single animal model provides a complete representation of the human CEP and caution should be taken when extrapolating data.^31^

### 3.10 Cellular regulation of CEP degeneration

With increasing degeneration of the CEP, its composition undergoes several changes that could reduce its permeability and limit nutrient transport. In tissue adjacent to degenerated discs, the calcium concentration was shown to be higher. There is a delicate balance between the nutrient demand and the nutrient supply, which is imposed by the cell population and controlled by the permeability of the CEP.^51^, ^54^ The classical paradigm is that any reduction in CEP permeability leads to nutrient retention and accumulation of lactate that in turn decreases the pH of the disc. A decrease in pH is directly related to hydrogen ion concentration. CEP permeability reduction or loss would impair disc oxygenation,^15^ and subsequently, reduce cell survivability and activity, being possibly a major regulator of IVD cell populations.^51^

Increased levels of calcium showed to enhance the cleavage of aggrecan by ADAMTS5.^192^ Not only was a decrease in aggrecan observed, but also a change in its composition changing from a 1:1 ratio of keratan sulfate to chondroitin sulfate to a 3:1 ratio.^71^ Those compositional changes result in a decrease in the net hydrophilic property of the tissue. Furthermore, a positive correlation between the degenerative state of the tissue and increased denaturation of type II collagen has been shown.^193^ Moreover, a mouse spondylosis model demonstrated that with increased age, apoptosis of chondrocytes in the CEP lead to a markable decrease in cell density. Subsequently, the disappearance of the CEP structure.^194^ This was corroborated in human CEP samples. In addition to decreased cell density and a higher rate of MC in degenerated CEP, expression of MMP3, MMP9, interleukin‐1 alpha (IL‐1α) and IL‐1β was increased.^195^ In addition, tissue inhibitors of metalloproteinases‐3 (TIMP3) also showed increased expression in degenerated CEP, suggesting a compensatory mechanism to regulate the increased ADAMTS expression. Interestingly, TIMP1 and TIMP2, but not TIMP3, were overexpressed in degenerated AF and NP compared with non‐degenerate tissues.^196^ CEP chondrocytes have also been demonstrated to have a different response than AC chondrocytes to the same stimuli.^197^ In response to hypertrophic stimuli such as Wnt agonist, CEP chondrocytes did not undergo the morphological changes seen in AC chondrocytes. However, they did show hypertrophic gene and protein expression and a decrease in proteoglycans. Oxidative damage‐induced stress was also shown to induce apoptosis and promote calcification in the human CEP.^198^ Neidlinger‐Wilke et al.^199^ showed in an in vitro model stimulating NP cells with conditioned media of CEPs a significant increase in IL‐6, IL‐8, and MMP3, as well as MMP13. Aggrecan and type II collagen were significantly decreased in NP cells exposed to the CEP‐conditioned media.^199^ Those findings indicate the interactions between the CEP and the NP tissue via molecular factors influencing the pathophysiology of disc degeneration. Limited studies have been performed on the expression of cytokines within the isolated CEP. In addition, there is strong evidence of genetic regulation of CEP cell fate through non‐coding RNA, which includes microRNA, short interference RNA and circular RNA. Proliferation, apoptosis, migration, and autophagy of CEP cells are the processes shown to be targeted by those RNAs and promoters of its degeneration.^200^, ^201^, ^202^, ^203^ There is also evidence for epigenetic roles in CEP degeneration regulation and in extension, IVD degeneration. Overexpression of histone methyltransferase enhancer of zeste homologue 2 (EZH2) in CEP cells produces reduced expression of *COL2*, *ACAN*, and *SOX‐9* genes and increased *ADAMTS5* and *MMP13* genes in rat CEP cells.^204^

Furthermore, reduced CEP permeability, and consequently reduced nutrition and lowered pH, can downregulate both catabolic and anabolic gene expression of the NP cells negatively affecting ECM homeostasis.^51^, ^54^ Specifically, the mRNA expression for *ACAN*, *COL2A1*, and *MMP2* in the NP reduces.^51^ Additionally, increased Ca^2+^ deposition leads to the activation of calcium‐sensing receptor (CaSR), causing increased catabolism through the suppression of collagen and GAG synthesis and can induce calcification of CEP tissue through upregulation of alkaline phosphatase (ALP).^192^ Further, deposition of Ca^2+^ in the IVD has been associated with increased parathyroid hormone‐related protein (PTHrP) signaling, which drives calcification.^205^

#### 3.10.1 Calcification and influence on permeability

Increased Ca^2+^ deposition in the CEP is seen with increased IVD degeneration in humans.^192^, ^205^ Calcification of the CEP is associated with decreased nutrient transport into the disc and waste transport out of the disc, which leads to nutrient starvation disc cells and a decreased pH within the disc, respectively.^2^ Further, calcification can lead to lower porosity, hydration, and permeability.^4^ With lower porosity and hydration, diffusion is impaired, and thus, dynamic loading has a greater effect on nutrient transport.^4^, ^94^ Static compression, however, reduces the CEP porosity and leads to reduced oxygen and greater lactate accumulation in the disc, limiting nutrient transport and gas exchange.^82^, ^94^ Altered nutrient transportation through the CEP has thus been suggested to be a significant factor in the pathogenesis of IVD degeneration.^206^ Preserving sufficient metabolite transport through the CEP is essential for the IVD to maintain its ECM and biochemical environment.^52^ Degenerative changes in the CEP could lead to up to 70% decrease in CEP permeability and, ultimately cell death.^9^ Overall, within calcified discs, dynamic compression improves disc nutrition while static compression impairs nutrition and leads to further degeneration. However, calcification is not always present in degenerated discs. When the CEP degenerates, GAG and collagen concentration decrease, which causes higher porosity and thus increased solute transport.^60^, ^207^ Dynamic compression has been shown to have less effect on higher porosity CEPs.^4^, ^54^ Nevertheless, the decreased matrix content also decreases the tensile modulus of the CEP, losing its ability to withstand mechanical forces.^60^

Degeneration has not been found to be correlated to GAG or water content separately, but rather to fixed charge density (FCD), which is a property related to both. Higher FCD hinders transport through creating steric and ionic barriers. It has been found to be directly proportional to IVD degeneration and inversely proportional to CEP permeability.^9^ This agrees with findings that CEPs with low permeability have high levels of collagen, aggrecan, and minerals which can physically block the solutes.^51^ Shirazi‐Adl et al.^50^ demonstrated that IVD cells start dying when CEP permeability decreases below 30% and the death rate increases exponentially as CEP permeability decreases further. The NP is the tissue most severely affected by CEP permeability changes.^10^ While the NP periphery is adjacent to the CEP, its center can be as far as 8–10 mm in the IVD.^51^ While there is also some diffusion through the outer AF, it is not enough to compensate for an impermeable CEP due to calcification.^4^

While calcification and dehydration have been shown to reduce the permeability of the CEP,^6^, ^51^ the effect of calcification in the CEP has also become a topic of debate recently. On one side, there is the hypothesis that calcification prevents fluid from flowing into the IVD to transport nutrients. Opposingly, there is the hypothesis that fluid movement, and thus the effect of calcification, has a negligible effect on nutrient transport. Supporters of the first hypothesis claim that calcification leads to a reduction of the pore volumes creating a physical impermeable barrier that obscures the fluid path.^6^ That, in turn, leads to reduced nutrient supply to the cells and, ultimately IVD degeneration.^6^, ^50^, ^54^ Supporters of the latter hypothesis claim that advection, or the movement of fluid, through the CEP has minimal effect on nutrient supply since the nutrient concentration in the IVD is controlled by diffusion. Diffusion occurs regardless of fluid velocity,^88^ especially when mechanical loading is present.^208^ Further, some studies claim that severe nutritional deprivation does not appear until calcification causes a 50% blockage.^10^ This level of blockage only occurs at late stages of degeneration, which contrasts other hypotheses that the depletion of CEP ECM might promote early degenerative mechanisms in the IVD through local cell starvation in the NP.^10^ The lack of blockage at early stages of IVD degeneration suggests that calcification as a physical barrier has minimal effect on IVD degeneration, however, cell catabolism induced by Ca^2+^ could induce IVD degeneration.^52^

Nonetheless, there is evidence that there is a strong positive correlation between CEP porosity and hydration.^4^, ^6^ Within extreme IVD degeneration, the CEP permeability barrier would be negligible due to the infiltration of blood vessels into the disc after damage. Therefore, both hypotheses could be correct depending on the age of the subject, degeneration level of the CEP, degree of calcification, and vascularization. This topic requires more intensive research to fully understand the effects of calcification on the IVD. Additionally, it is important to consider the poromechanical interactions among the BEP, the CEP and the NP to understand the mechanotransport of nutrients.^10^

## 4 EMERGING THERAPIES

Nutritional supply controls the population and activity of the IVD cells to synthesize and maintain the ECM. Thus, for any disc cell therapy to succeed, maintenance of the nutrition supply is crucial. Furthermore, even a successful attempt to biologically repair the IVD can fail in the long run if the nutrition‐population balance is not maintained.

Most therapies aim to surgically remove the source of LBP or re‐establish IVD mechanical functions overlooking the sustainability of such operations, which is part of why they have a low success rate.^51^ Intradiscal biological therapy is a non‐invasive alternative that consists of injecting genes, growth factors, and other molecules to the IVD that aim to boost the cell population and produce ECM to restore a physiological environment.^51^ Yet, increasing the cell number without increasing the nutrient supply is unsustainable because it will lead to the supply–demand disturbance, as previously mentioned.^51^ A FE analysis study demonstrated that cell injection could lead to increased and accelerated degeneration in the IVD due to a higher supply–demand disturbance that is directly related to the state of the CEP, that is, whether it is healthy, calcified, or thinned.^54^ Thus, the harsh nutrient environment of the IVD, particularly concerning the state of the CEP, must be accounted for to make any cell therapy beneficial. They suggested reducing the CEP thickness to enhance nutrition in the IVD, but this could compromise the mechanical stability of the IVD.

Treatments targeting the CEP are often focused on enhancing permeability. One possible method would be decalcification of the CEP through injecting compounds that can bind calcium.^209^ Other methods suggested include enzymatic treatment, such as trypsin or hyaluronidase, to remove large proteoglycans from the CEP.^209^ However, chemical injection with enzymes is also used to induce degeneration in animal models.^180^ Therefore, the use of enzymes for CEP therapies should be strictly restricted to the CEP. Excessive/untargeted protease activity as in antiquated chemonucleolysis can even trigger MC within 6 weeks.^210^ Dolor et al.^207^ treated the CEP with MMP8 to reduce the matrix and enhance solute uptake and nutrient diffusion. MMP8 is selective for type II collagen and aggrecan, which are the two main matrix components of the CEP. However, these are also the main components of the NP, and therefore it is crucial that using MMP8 or another enzyme does not induce degeneration within the NP or AF due to off‐target digestion or matrix fragments triggering a catabolic response. One method Dolor et al.^207^ considered to avoid this was using targeted delivery through injection and linking the enzyme to bulky nanoparticles that cannot migrate to other tissues. Nevertheless, this treatment was only performed in human cadaveric CEPs, so it is unknown whether a catabolic response will be produced testing in vivo.

Although therapies addressing the CEP are still preliminary, studies have shown that incorporating the CEP into a tissue engineered disc improves the performance.^211^ While development of functional NP and AF replacements is important, these tissues must be integrated into the CEP to allow for successful and functional IVD replacement.^209^, ^212^ Studies have shown that using direct contact co‐culture of AF^212^ or NP cell‐seeded scaffolds^213^ with chondrocyte‐seeded scaffolds produced native interface characteristics. However, although type I collagen, type II collagen, and aggrecan distribution were like native tissue, the apparent mechanical strength was 57‐times weaker than in native tissue segments, which means it would not function well under daily mechanical loads.^212^ Obtaining comparable mechanical properties of a native disc has been a large problem in tissue engineered scaffolds.^214^, ^215^ Gullbrand et al.^211^ have tested endplate‐modified disc‐like angle ply structures (eDAPS) as replacement discs in rat and goat animal models. In the eDAPS, the endplate was made up of acellular, porous polyE‐caprolactone (PCL) foams which was combined with the NP and AF components.^211^ They showed that after 20 weeks with external fixation, native cells from neighboring tissues could migrate into the CEP structure and start producing matrix components and sparse vascularization,^211^ which is a focus area of CEP treatments.^209^ However, it should be noted that while vascularization is important for the BEP and CEP, it can lead to increased degeneration and pain if angiogenesis occurs in the NP.^216^ Although Gullbrand et al.^211^ observed improvements in tensile properties, the failure strain of the eDAPS was only 50% that of native values. However, it was shown that the constructs which included a CEP structure outperformed those without.^217^

Bioprinting is also a popular technique for developing tissue engineered constructs but has the same issue of sub‐optimal mechanical properties.^214^ Printing using bioinks with reinforcement structures such as carbon fibers or alumina platelets has been considered for recreating load‐bearing tissues such as the CEP, although low printing resolutions can limit the functionality.^214^ Using decellularized ECM is another option for tissue engineered constructs, which addresses the problem of low printing resolutions, and could help with the design of 3D printed scaffolds.^215^ Nevertheless, mechanics of chemically modified decellularized ECM also have weak mechanical properties that do not approach the Young's modulus of native tissues.^215^ Further, scalability and reproducibility alongside high manufacturing costs are limiting in the bio fabrication of IVD constructs.^214^

Recently, Liu et al.^21^ have identified the presence of progenitor cells in the CEP. After culturing in agarose, cells isolated from degenerated human CEPs were found to be positive for stem cell markers *OCT‐4*, *NANOG*, *and SOX‐2* as well as common BM‐MSCs markers *CD105*, *CD73*, *CD90*, *CD44*, *CD166*, and *Stro‐1*.^21^ Further, the group found that NP cells that were stimulated with CEP progenitor cells isolated from healthy subjects showed a decrease in apoptotic rate by releasing exosomes that activate the PI3K/AKT signaling pathway.^218^ Thus, CEP progenitor cell‐derived exosomes could be a possible therapeutic tool in the treatment of IVD degeneration.

Several studies have also found potential targets for the treatment of the CEP with regard to IVD degeneration, although additional research needs to be done before testing. For example, the HIF1A/MIF pathways has been shown to play a role in promoting chondrogenesis, while also inhibiting osteogenesis.^219^ EZH2 inhibition has also shown promise as a therapeutic target to combat CEP degeneration through upregulation of SOX9.^204^ Managing oxidative stress and damage could be another novel therapy target. For this, research has found inhibiting ROS reduced apoptosis in CEP cells under oxidative stress^198^ and, similarly, enhancing the NrF/Keap1 pathway in CEP cells increases antioxidants that can combat damage from ROS.^220^ Certain microRNAs (miRNAs), specifically miR‐495‐3p^202^ and miR‐34a,^201^ have been found to play a role in ECM degradation and chondrocyte apoptosis, respectively. Therefore, silencing these miRNAs could be a novel treatment for CEP and IVD degeneration.

## 5 CONCLUSION

The CEP is a unique tissue distinct from other cartilaginous tissues in morphology, gene expression, and mechanical and transport properties. It is an essential component of the IVD and is considered to play a key role in the early stages of IVD degeneration due to its fundamental role in nutrient transport.^10^, ^221^ However, most data regarding IVD degeneration have focused on the NP and AF tissues. Further, research that does include the CEP often does not distinguish it from the BEP. New MRI techniques^114^, ^116^ as well as the standardization of histopathology scoring in the CEP^74^ allow for the characterization of the CEP separate from the IVD or BEP, and thus future research should aim to investigate the CEP as an independent tissue type. In particular, the role of the CEP in MC should be investigated further as most research is limited to the BEP or the combined BEP and CEP.

Additionally, the CEP is often considered to be the same as AC particularly in modeling and simulations.^10^ However, as detailed in this review, the CEP differs from AC in function, cellular response, biochemical content, and material properties.^9^, ^24^, ^60^, ^65^, ^66^, ^67^, ^89^, ^197^ Thus, future research should aim to characterize the CEP itself, and avoid assumptions that the CEP will behave and/or respond the same as AC.

Much is still unknown about the mechanics and transport properties of the CEP, and reported values show a large variation. The wide range of reported values is due to various testing methods, environmental conditions, species, and degree of degeneration,^4^, ^10^, ^52^, ^53^, ^60^ and thus highlights the need for standardized, reproducible methods and guidelines for investigating the CEP. Likewise, the authors recommend to standardize specifying the terms cartilaginous endplate (CEP) and bony endplate (BEP) and advise avoiding the term vertebral endplate. Further, it is essential that researchers clearly state which tissue is being investigated, whether it is CEP, BEP, or a combination of both.

Calcification should also be a focus of future research, as there is controversy regarding the role it plays in CEP permeability. It is accepted that calcification occurs in degeneration. However, it is unclear whether disc damage occurs at an early stage due to impaired nutrient transport induced by calcification, cellular level changes caused by excess Ca^2+^ in the environment,^6^, ^10^, ^51^, ^52^, ^88^, ^208^ and/or due to early depletion of the CEP ECM (possibly related with MC) disrupting the functional fluid exchange between the vertebrae and NP under mechanical loads.^10^

Overall, much is still unknown about the CEP and the mechanisms of CEP degeneration. Additional research is needed to elucidate the mechanical and transport properties, gene expression, cellular response, and how these traits change with degeneration and age. Understanding the CEP is essential to develop therapies that target or include the CEP. Notably, the CEP should be considered in any treatment of the IVD, as the nutrient and waste transport must be functional for any therapy targeting the NP or AF to be successful. Thus, any long‐lasting and sustainable therapy aiming to reverse IVD degeneration should target the CEP first or simultaneously with the NP and AF to rescue the IVD from a pathological environment.

## AUTHOR CONTRIBUTIONS

Katherine B. Crump, Ahmad Alminnawi, Liesbet Geris, Christine Le Maitre, and Benjamin Gantenbein contributed to the main conception and design of this review. Katherine B. Crump, Ahmad Alminnawi, Paola Bermudez‐Lekerika, Roger Compte, Francesco Gualdi, Terence McSweeney, Estefano Muñoz‐Moya, Andrea Nüesch, Stefan Dudli, and Christine Le Maitre contributed to the literature review and drafted the text. Liesbet Geris, Stefan Dudli, Jaro Karppinen, Jérôme Noailly, Christine Le Maitre, and Benjamin Gantenbein provided scientific guidance. Liesbet Geris, Jaro Karppinen, Jérôme Noailly, Christine Le Maitre, and Benjamin Gantenbein sourced funding. All authors edited the text and approved the final version of the manuscript. Katherine B. Crump, Ahmad Alminnawi, Paola Bermudez‐Lekerika, Terence McSweeney, Andrea Nüesch, and Christine Le Maitre contributed to the figures.

## CONFLICT OF INTEREST STATEMENT

Benjamin Gantenbein and Christine Le Maitre are editorial board members of JOR Spine and co‐author of this article. They were excluded from editorial decision‐making related to the acceptance of this article for publication in the journal. All other authors have no conflicts of interest to declare in relation to this article.
